# Attitude and Practice of Folic Acid Consumption Among Saudi Pregnant Women at Yanbu City, Kingdom of Saudi Arabia

**DOI:** 10.7759/cureus.28860

**Published:** 2022-09-06

**Authors:** Shorooq Al-Marwani, Ahlam Al-Zahrani

**Affiliations:** 1 Nursing, Yanbu General Hospital, Ministry of Health, Yanbu, SAU; 2 Department of Maternity and Child, Faculty of Nursing, King Abdulaziz University, Jeddah, SAU

**Keywords:** kingdom of saudi arabia, pregnant females, folic acid, practice, attitude

## Abstract

Background

Folic acid (FA) intake is important for pregnant women for the healthy growth and development of their babies. It is well known that the intake of FA before and during pregnancy help in reducing the risks of congenital anomalies.

Methods

A quantitative cross-sectional design was used to assess pregnant women's attitudes and practices related to FA. This study was conducted in the Antenatal Clinic at Yanbu General Hospital from February 2021 to May 2021. Structured questionnaires were used for data collection from a sample of pregnant women (n=65).

Results

The study revealed that less than half of the study sample consumed FA daily and only 46.2% of women started consuming FA during the first month of pregnancy.

Conclusion

Action should be taken by the Saudi Health Organization for empowering child-bearing women to consume adequate sources of FA at the appropriate time and frequency.

## Introduction

Folic acid (FA) intake is important for pregnant women for the healthy growth and development of their babies. It is well known that the intake of FA before and during pregnancy helps in reducing the risks of congenital anomalies. FA reduces homocysteine levels in the blood. In the 1930s-1940s, Lucy Willis, a physician, discovered FA supplements after conducting anemia-based tests among women in India [1]. Spina bifida is particularly common in the Kingdom of Saudi Arabia (KSA), affecting 1.2 in 1000 people [2]. Improvements in pregnancy outcomes reduce neural tube defects (NTDs), morbidities, and mortalities involving neonates or their mothers.

Pregnant women attending antenatal clinics can access knowledge from nurses, doctors, and gynecologists regarding the intake, use, dosage, and required administration of FA [3]. Moreover, healthcare professionals play a critical role in the intake of FA to enhance pregnancy outcomes for women under their care. Getting the right dosage of FA is crucial for pregnant women. It was reported that pregnant women should take 400 µg of folate per day during the periconceptional period [4]. Using FA supplements before and after pregnancy is imperative to mitigate the risk of preterm births and small for gestational age (SGA). Dietary folate intake should complement the supplements before and after the pregnancy period.

Different scholars have assessed the knowledge of FA among females. A study with 96 participants showed a low intake of FA, as only 16%, and 7% admitted the consumption of multivitamins and FA supplements, respectively [5]. According to the researchers, at least 71% of the learners consumed multivitamins during pregnancy while 54% understood food sources of FA. The research showed an understanding of FA and its impact on pregnancy, including the reduction of neural tube defects (NTDs), among the participants.

The outcomes of questionnaire-based cross-sectional studies are aligned with the findings [6,7]. Both studies agreed on the need for increasing the use of FA among females in KSA. A sample of 254 Saudi pregnant women from the Family Medicine Department revealed that only 22% of the pregnant women used it before conception [6]. Additionally, only 50% of the women understood the exact reason for FA diet or supplementation during pregnancy. This indicated the need for an increased level of awareness among Saudi women on FA supplementation during the preconception period.

The level of awareness of FA supplementation or diet intake during preconception varies from region to region. A study using a sample of 300 childbearing married women aged 19-45 years in the Hail region of KSA found that 84% used FA during different pregnancy phases [8]. A Saudi study has reported that the majority of their study sample had a poor total score on knowledge about FA, which may negatively affect FA consumption [9]. Based on the literature review undertaken, no studies have been conducted in relation to FA attitude and practice in Yanbu City, KSA. This could be due to the fact that Yanbu is a small city, which may hinder researchers from conducting their research there. Therefore, the current study aims to assess Saudi pregnant women’s attitudes and practices in Yanbu City, KSA.

## Materials and methods

Study design 

One of the essential steps in conducting a research study is selecting a suitable design. The design helps the researcher achieve the research aim and objectives. In the current study a quantitative, cross-sectional design was applied to assess the attitudes and practices related to FA consumption among pregnant women in Yanbu City. This type of design helps the researchers to examine the relationships and/or differences among variables.

Setting

The research setting is the physical, social, and cultural location where a study is conducted. This study was conducted at Yanbu City in KSA, which is a small and beautiful city located in the Western province of KSA. Data collection started in February 2021 and lasted until May 2021 in the Antenatal Clinic at Yanbu General Hospital (YGH). YGH was selected by the researchers because it is the largest hospital in Yanbu City and is the only governmental hospital in this region. In addition, it has a good capacity for maternal cases, which could help obtain the target population for the study. The working hours in the clinic are 08:00 AM to 04:00 PM, 5 days a week. The researcher visited the antenatal clinic daily for data collection during the period.

Sampling

The sampling design involved the selection of the subjects as study samples from the population under study. A purposeful sampling method was used to select the study participants in this study. The population of this study included (a) Saudi pregnant women attending the Antenatal Clinic at YGH; (b) aged 18-45 years; (c) living in Yanbu City; (d) willing to participate in the study. A total of 65 pregnant women were included in the current study.

Study tool

The tool of this study was a structured questionnaire developed by the researchers concerning the comprehensive reading of the related literature, and it was then revised by two academic professors specializing in maternity health in the Nursing Faculty at KAU. The questionnaire was piloted on seven selected women to assess the clarity and the time needed to fill it. In addition, reliability was tested using Cronbach's Alpha (α) test, which confirmed that the scales used in the questionnaire items are reliable. The questionnaire consisted of three parts: i) Demographic data and obstetric history; ii) Attitude toward FA; iii) Practice toward FA. The structured questionnaires were filled out by the study participants when they were in the clinic waiting area. Those who could not read or write were supported by other women who visited the clinic to help them in filling the questionnaire.

Data analysis

The questionnaire was coded and analyzed using Statistical Package for Social Sciences (SPSS) version 25 (IBM Corp., Armonk, NY). Data were presented in tables in the form of frequencies and percentages. A Chi-square test of significance with a 95% confidence level was used to find the association between variables. The attitude was classified as positive or negative. A positive attitude was defined as the study participants preferring to consume FA from both sources, natural and supplements. Practice was scored as excellent (>60.0%), good (60-40%), and poor (<40.0%). Excellent practice was defined when the study participants took FA in the first trimester of their current pregnancy, daily, and started to take it three months before their pregnancy.

Ethical considerations

This study was approved by the Ethical Committee of the Nursing Faculty at King Abdulaziz University (KAU) in Jeddah KAU (Ref. No. 1M.10) and then by the ethical committee in the Ministry of Health (MOH) in Madinah (IRB 555). The researcher explained the purpose and aim of the study to the women participants. The researcher obtained verbal consent from the study participants, who were informed that they have the right to withdraw from the study at any point in time.

## Results

Participant's socio-demographic data

Table 1 represents the socio-demographic data of the study participants according to their age, education, marital status, income, residence, and working status. Thirty point eight percent (30.8%) of the study sample were aged 26-30 years, 26.2% of them are aged between 18 and 25, while 16.9% of them are from 36-45 years old.

**Table 1:** Participant's socio-demographic data

Variables	Number	Percentage (%)
Participant’s age groups		
18–25 years	17	26.2
26–30 years	20	30.8
31–35 years	17	26.2
36–45 years	11	16.9
Education		
Read and Speak Arabic	16	24.6
Secondary	15	23.1
University	32	49.2
Others	2	3.1
Marital status		
Married	59	90.8
Separated/Divorced	6	9.2
Income		
3000–6000 SAR	26	40.0
7000–9000 SAR	15	23.1
More than 9000 SAR	24	36.9
Residence		
North	4	6.2
South	3	4.6
East	2	3.1
West	56	86.2
Participants’ working status		
Housewife	35	53.8
Student	10	15.4
Working	20	30.8

The majority of the study participants (90.8%) are married while 9.2% are separated or divorced. Forty percent of the participants have an income between 3000 and 6000 SAR and 36.9% of them have an income of more than 9000 SAR. The majority of the samples (86.2%), are from the West while 6.2% of them originate from the North. Regarding educational qualifications, 49.2% of the participants have a university degree while 23.1% have a secondary school education. In addition, more than half (53.8%) of the participants are housewives while 30.8% are working.

Participant's medical and obstetric history

Table 2 shows that the majority of the participants (67.7%) did not have any history of diseases while 23.1% had anemia. The gestational age of 64.6% of participants was 1-3 months, and 23.1% of them were 4-6 months. In addition, 76.9% of the participants had one to three pregnancies while 23.1% of them had four to six pregnancies. Half of the study participants (50.8%) had one to three deliveries while 26.2% of them did not have any delivery. In addition, 60.0% of the participants had a previous normal vaginal delivery while 13.8% of them had a previous cesarean section delivery. In addition, the majority of the participants (93.8%) did not have congenital anomalies in their previous births. More than half of the participants (52.3%) had a plan for the current pregnancy while 47.7% did not.

**Table 2:** Participant's medical and obstetric history

Variables		Number			Percentage (%)	
History of medical diseases						
No history of diseases	44			67.7		
Anemia	15			23.1		
Diabetes mellitus	3			4.6		
Hypertension	1			1.5		
Others	2			3		
Gestational age						
1–3 months			42			64.6
4–6 months			15			23.1
7–9 months			8			12.3
Gravida						
1–3 pregnancies			50			76.9
4–6 pregnancies			15			23.1
Para						
0 deliveries			17			26.2
1–3 deliveries			33			50.8
4–6 deliveries			15			23.1
Abortion						
0 times			43			66.2
1–3 times			22			33.8
Mode of previous deliveries						
No previous deliveries			17			26.2
Normal vaginal delivery			39			60.0
Cesarean section			9			13.8
Previous congenital anomalies						
No			61			93.8
Yes			4			6.2
Planning for current pregnancy						
Yes			34			52.3
No			31			47.7

Participants' attitudes toward FA

Table 3 shows that the participants have a positive attitude in relation to FA consumption. Forty-seven point seven percent (47.7%) of the study participants prefer to take FA from natural sources only, 53.8% of them prefer to take FA from complementary sources only while 80.0% prefer to take FA from both natural and complementary sources.

**Table 3:** Participants' attitudes toward FA FA: folic acid

Attitude statements	No.	Yes
I prefer to take FA from natural sources only.	34 (52.3%)	31 (47.7%)
I prefer to take FA from complementary sources only.	30 (46.2%)	35 (53.8%)
I prefer to take FA from both natural and complementary sources.	13 (20.0%)	52 (80.0%)

Participants' practices of FA

Table 4 shows that the majority (92.3%) of the participants took FA in their current pregnancy. However, only 31.7% took FA daily in their current pregnancy. In addition, 46.2 % of the participants started to take FA in the first month of their pregnancy while 30.7 % of them started to take it three months before their pregnancy. In addition, 3.1% of the participants did not take FA during this pregnancy because they did not care about it, 1.5% of them did not take it because they take it from natural sources only, and another 1.5% did not take it because they forgot to take it.

**Table 4:** Participants' practices of FA FA: folic acid

Practice	Answer	Frequency	%
Took FA in their current pregnancy.	Yes	60	92.3
	No	5	7.7
Took FA daily in their current pregnancy.	Yes	19	31.7
	No	41	68.3
Starting time of taking FA.	In the first months	30	46.2
	Three months before pregnancy	20	30.7
	Cannot remember	3	1.95
	Others	7	10.7
	Did not take FA	5	7.7
Causes of not taking FA during pregnancy.	Forgot	1	1.5
	Take it from natural sources only	1	1.5
	I do not care	2	3.1
	Others	1	1.5
Causes of not taking FA three months before pregnancy.	Did not know the importance of it	16	26.6
	Did not plan for pregnancy	11	18.3
	Others	8	13.3
The one who provided information about FA.	Physician	47	72.3
	Nurse	2	3.1
	Yourself	11	16.9
	Did not take FA	5	7.7
Method of receiving FA.	Prescription	42	64.6
	Pharmacy	18	27.7

Moreover, 26.6 % of the participants did not take FA three months before their pregnancy because they did not know its importance, 18.3% of them did not take it because they did not plan for the pregnancy while 13.3 % of them did not take FA three months before pregnancy due to other causes. The majority of the study sample received information about FA from their physician (72.3). While 64.6% of them took FA through a physician’s prescription and 27.7% of them took it by buying it from a pharmacy.

Relation between taking FA in current pregnancy and socio-demographic data

Table 5 shows that there is a significant association between taking FA in the current pregnancy and the participants’ income. The post hoc test showed that with a higher level of income, the possibility of taking FA increases (p<0.01); 95.8% of the participants having an income of more than 9,000 SAR took FA while only 4.2% of them did not. Moreover, 73.3% of those having an income between 7,000 and 9,000 SAR took FA compared to 26.7% who did not. On the other hand, there is no significant association between taking FA in the current pregnancy and the participants’ age, residence, marital status, education, and employment (p>0.05).

**Table 5:** Relation between taking FA in current pregnancy and socio-demographic data FA: folic acid

Taking FA in the current pregnancy					Chi-square x2	p-value
Demographic factors	No	%	Yes	%		
Participants’ age						
18–25 years	1	5.9	16	94.1	3.847	0.279
26–30 years	0	0.0	20	100.0		
31–35 years	2	11.8	15	88.2		
36–45 years	2	18.2	9	81.8		
Residence						
North	0	0.0	4	100.0	0.871	0.833
South	0	0.0	3	100.0		
East	0	0.0	2	100.0		
West	5	8.9	51	91.1		
Marital status						
Married	5	8.5	54	91.5	0.551	0.458
Separated/Divorced	0	0.0	6	100.0		
Education						
Intermediate	1	6.2	15	93.8	0.363	0.948
Secondary	1	6.7	14	93.3		
University	3	9.4	29	90.6		
Others	0	0.0	2	100.0		
Employment						
Housewife	3	8.6	32	91.4	0.317	0.853
Student	1	10.0	9	90.0		
Working	1	5.0	19	95.0		
Income						
3,000–6,000 SAR	0	0.0	26	100.0	10.192	0.006
6,000–9,000 SAR	4	26.7	11	73.3		
More than 9,000 SAR	1	4.2	23	95.8		

Relation between taking FA three months before pregnancy and socio-demographic data

Table 6 shows the participants’ practices and demographic data. It shows that there is a significant relation between taking FA three months before pregnancy and the participants’ employment status. Housewives tend to take FA three months before pregnancy significantly more than working women (p<0.05). In addition, there is a significant association between taking FA three months before pregnancy and the participants’ education. With more educational qualifications, the tendency to take FA three months before pregnancy decreases (p<0.05); 65.6% of the participants who have a university degree did not take FA three months before pregnancy compared to 34.4% who did.

**Table 6:** Relation between taking FA three months before pregnancy and socio-demographic data FA: folic acid

Taking FA three months before pregnancy					Chi-square x2	p-value
Demographic factors	No	%	Yes	%		
Participants’ age						
18–25 years	16	94.1	1	5.9	5.734	0.125
26–30 years	15	75.0	5	25.0		
31–35 years	11	64.7	6	35.3		
36–45 years	10	90.9	1	9.1		
Residence						
North	3	75.0	1	25.0	1.942	0.585
South	3	100.0	0	0.0		
East	1	50.0	1	50.0		
West	45	80.4	11	19.6		
Marital status						
Married	46	78.0	13	22.0	1.653	0.199
Separated/Divorced	6	100.0	0	0.0		
Education						
Intermediate	16	100.0	0	0.0	10.924	0.012
Secondary	14	93.3	1	6.7		
University	21	65.6	11	34.4		
Others	1	50.0	1	50.0		
Employment						
Housewife	20	57.1	15	42.9	16.300	0.000
Student	9	90.0	1	10.0		
Working	16	80.0	4	20.0		
Income						
3,000–6,000 SAR	25	96.2	1	3.8	11.699	0.003
6,000–9,000 SAR	13	86.7	2	13.3		
More than 9,000 SAR	14	58.3	10	41.7		

Furthermore, there is a significant relation between taking FA three months before pregnancy and the participants’ income. With more income, the tendency to take FA three months before pregnancy decreases (p<0.01); 58.3% of the participants who have an income of more than 9000 SAR did not take FA three months before pregnancy compared to 41.7% who did. On the other hand, there is no significant association between taking FA three months before pregnancy and the participants’ age, residence, and marital status (p>0.05).

Relation between taking FA daily and socio-demographic data

Table 7 shows the relation between the participants’ practices and socio-demographic factors. There is no significant association between taking FA daily and the participants’ age, residence, marital status, educational level, income, and employment (p>0.05).

**Table 7:** Relation between taking FA daily and socio-demographic data

	Took FA in current pregnancy daily					Chi-square x2	p-value
Demographic factors		No	%	Yes	%		
Participants’ age							
18–25 years		12	70.6	5	29.4	1.840	0.606
26–30 years		15	75.0	5	25.0		
31–35 years		13	76.5	4	23.5		
36–45 years		6	54.5	5	45.5		
Residence							
North		3	75.0	1	25.0	2.905	0.407
South		1	33.3	2	66.7		
East		2	100.0	0	0.0		
West		40	71.4	16	28.6		
Marital status							
Married		43	72.9	16	27.1	1.378	0.240
Separated/Divorced		3	50.0	3	50.0		
Education							
Intermediate		11	68.8	5	31.2	3.748	0.290
Secondary		13	86.7	2	13.3		
University		20	62.5	12	37.5		
Others		2	100.0	0	0.0		
Employment							
Housewife		24	68.6	11	31.4	0.499	0.779
Student		8	80.0	2	20.0		
Working		14	70.0	6	30.0		
Income							
3,000–6,000 SAR		19	73.1	7	26.9	1.478	0.478
6,000–9,000 SAR		12	80.0	3	20.0		
More than 9,000 SAR		15	62.5	9	37.5		

Relation between the attitude of taking FA from a natural source only and socio-demographic data

Table 8 shows the relationship between the participants’ attitudes and socio-demographic data. It shows that there is no significant association between the preference to take FA from natural sources only and the participants’ age, residence, income, educational level, and employment status (p>0.01).

**Table 8:** Relation between the attitude of taking FA from a natural source only and socio-demographic data

Prefer to take FA from natural sources only					Chi-square x2	p-value
Demographic factors	No	%	Yes	%		
Participants’ age						
18–25 years	9	52.9	8	47.1	2.457	0.483
26–30 years	9	45.0	11	55.0		
31–35 years	8	47.1	9	52.9		
36–45 years	8	72.7	3	27.3		
Residence						
North	2	50.0	2	50.0	2.271	0.518
South	2	50.0	2	50.0		
East	2	100.0	0	0.0		
West	29	51.8	27	48.2		
Marital status						
Married	31	52.5	28	47.5	0.014	0.905
Separated/Divorced	3	50.0	3	50.0		
Education						
Intermediate	5	31.2	11	68.8	4.187	0.242
Secondary	8	53.3	7	46.7		
University	20	62.5	12	37.5		
Others	1	50.0	1	50.0		
Employment						
Housewife	17	48.6	18	51.4	2.457	0.483
Student	7	70.0	3	30.0		
Working	10	50.0	10	50.0		
Income						
3,000–6,000 SAR	11	42.3	15	57.7	3.217	0.200
6,000–9,000 SAR	7	46.7	8	53.3		
More than 9,000 SAR	16	66.7	8	33.3		

Relation between the attitude of taking FA from a complementary source only and socio-demographic data

Table 9 shows the relation between the participants’ attitudes and socio-demographic data. It shows that there is no significant association between the participants’ preference to take FA from complementary sources only and their age, residence, income, educational level, and employment status (p>0.01).

**Table 9:** Relation between the attitude of taking FA from a complementary source only and socio-demographic data FA: folic acid

Prefer to take FA from complementary sources only					Chi-square x2	p-value
Demographic factors	No	%	Yes	%		
Participants’ age						
18–25 years	8	47.1	9	52.9	3.774	0.287
26–30 years	6	30.0	14	70.0		
31–35 years	9	52.9	8	47.1		
36–45 years	7	63.6	4	36.4		
Residence						
North	1	25.0	3	75.0	3.254	0.354
South	1	33.3	2	66.7		
East	2	100.0	0	0.0		
West	26	46.4	30	53.6		
Marital status						
Married	28	47.5	31	52.5	0.437	0.508
Separated/Divorced	2	33.3	4	66.7		
Education						
Intermediate	7	43.8	9	56.2	3.553	0.314
Secondary	5	33.3	10	66.7		
University	16	50.0	16	50.0		
Others	2	100	0	0.0		
Employment						
Housewife	14	40.0	21	60.0	0.437	0.508
Student	4	40.0	6	60.0		
Working	12	60.0	8	40.0		
Income						
3,000–6,000 SAR	11	42.3	15	57.7	0.299	0.861
6,000–9,000 SAR	7	46.7	8	53.3		
More than 9,000 SAR	12	50.0	12	50.0		

Relation between the attitude toward taking FA from both sources and socio-demographic data

Table 10 shows the relationship between participants’ attitudes and demographic data. It shows that there is no significant association between the participants’ preference to take FA from natural and complementary sources and their age, residence, income, educational level, and employment status (p>0.01).

**Table 10:** Relation between the attitude of taking FA from both sources and socio-demographic data FA: folic acid

Prefer to take FA from natural and complementary sources					Chi-square x2	p-value
Demographic factors	No	%	Yes	%		
Participants’ age						
18–25 years	3	17.6	14	82.4	5.720	0.126
26–30 years	1	5.0	19	95.0		
31–35 years	6	35.3	11	64.7		
36–45 years	3	27.3	8	72.7		
Residence						
North	0	0	4	100	1.905	0.592
South	1	33.3	2	66.7		
East	0	0	2	100		
West	12	21.4	44	78.6		
Marital status						
Married	12	20.3	47	79.7	0.046	0.830
Separated/Divorced	1	16.7	5	83.3		
Education						
Intermediate	2	12.5	14	87.5	1.549	0.671
Secondary	4	26.7	11	73.3		
University	7	21.9	25	78.1		
Others	0	0	2	100		
Employment						
Housewife	7	20.0	28	80.0	0.000	1.000
Student	2	20.0	8	80.0		
Working	4	20.0	16	80.0		
Income						
3,000–6,000 SAR	3	11.5	23	88.5	2.841	0.242
6,000–9,000 SAR	5	33.3	10	66.7		
More than 9,000 SAR	5	20.8	19	79.2		

## Discussion

This current study assesses the attitude and practice regarding FA consumption among women of childbearing age in Yanbu City, KSA. It concluded that, generally, there is a positive attitude and slightly good practice regarding FA consumption in the most vulnerable group, which is women of childbearing age. Despite the fact that the majority of the study sample prefer to take FA from both natural and supplementation sources, it was found that more than half of the participants preferred to take FA as a supplement only and a few others preferred it in the form of food rich in folate. The outcomes are congruent with the results of another study [10]. This indicates that some participants need to be educated about the importance of both sources of FA during pregnancy.

The majority of the participants of this current study used FA during pregnancy. This finding agrees with that of another study conducted in Riyadh, KSA, which found that most of the study participants took FA during pregnancy [6]. In addition, a study found that women aged 18-60 years in Riyadh, KSA, used dietary supplementation of FA [11]. Another study also reported similar findings [12]. In contrast, a study conducted in India reported that the use of FA among pregnant women was low, which consequently led to an increase in NTDs [13]. The reason for increased FA consumption by pregnant women in the current and other Saudi studies could be that KSA health policy strictly encourages FA intake during antenatal care visits. It is worth mentioning that Saudi governmental hospitals routinely advise pregnant women to take FA and give free FA supplements. Currently, in KSA, there is an increase in empowering women's education and involvement in the workforce, which can likely attribute to better FA use.

Regarding the daily use of FA during pregnancy, the current study revealed that less than half of the study sample consumed FA daily. The same result was observed in China, which found that less than 50% of women consumed FA regularly [14]. The possible reason for this could be that the women didn't have adequate information about the proper time of FA intake or they forget to take it. On the contrary, a study revealed that 80% of the study sample used FA daily, and they found a relationship between the educational level and daily consumption of FA [15].

A study has associated educational sessions with the elimination of knowledge gaps created by societies owing to negative beliefs about the effectiveness of FA supplements besides natural sources of vitamins [16]. This study's findings are supported by another study that found increased use of FA supplements in 55.8% of the 360 studied pregnant women in Riyadh following interactions with pharmacists [17]. It can be thus argued that education is important for providing opportunities to positively influence individual knowledge, awareness, and attitudes. Therefore, health care providers should emphasize providing women with focused education, particularly during the premarital and antenatal periods, in order to improve their attitudes and practice concerning FA. However, a study conducted in KSA found that 77% of pregnant women with epilepsy took FA daily [18]. In this study, no relation between educational level and daily intake of FA was found.

Regarding FA use three months before pregnancy, a low percentage of the current sample took FA three months before conception. The outcomes are consistent with the finding of another study in KSA [6]; they reported that only 10% of the study participants took FA three months before conception. The present study found a significant relation between taking FA three months before pregnancy and the participants' employment status. It was found that a significant number of housewives took FA three months before pregnancy than working women. This could be because of the busy routine of working women. Another reason could be that housewives plan their pregnancies better as compared with working women. However, this reason was not investigated in this study and thus needs further exploration.

Similarly, a study found that 41.5% of their study samples took FA during the preconception period while 47.4% took it during pregnancy [19]. This result was found to be associated with the education level, planning for pregnancy, and the pregnancy experience. However, various studies have found that most participants took FA before pregnancy [20,21].

The current data shows that 46.2% of women started consuming FA during the first month of pregnancy. A study found a relatively similar result [22]; more than half of the study sample consumed FA during the first month of pregnancy. It can be argued that although the study participants of the current study used FA during pregnancy, the time and frequency of use were not as per the recommended standard, which limited the benefits of FA use. Insufficient FA can increase the risk of NTDs and folate deficiency anemia; it occurs in the early phase of pregnancy before the mother probably even realizes that she is pregnant. Therefore, the daily use of at least 400 μg of FA three months before conception, and the first three months of pregnancy is important in order to protect the health of mothers and their babies.

The current study has some limitations that should be considered in future studies. The generalizability of the current study was affected. First, the study sample was small this could be because the study was conducted during the COVID-19 pandemic, which may limit the number of pregnant women who visited the clinic and accepted to participate in the study. Second, this study was exclusively on women visiting YGH outpatient clinics. Therefore, further studies should be conducted with large sample sizes and various geographical settings to establish the generalizability of the result. In addition, it is worth mentioning that this study did not investigate the participant's diets and beverages they consumed daily in order to assess their actual FA consumption, which would be good for further research.

## Conclusions

FA is the most important vitamin taken during pregnancy. The results of this study are in line with other national and international studies in relation to FA consumption by pregnant women. The study participants had a positive attitude and slightly good practice in relation to FA consumption. Income and employment status have an influence on FA attitude and practice. The majority of the participants took FA in their current pregnancy, however, more than half of them did not take it daily in their current pregnancy and half of them did not take FA three months before their pregnancy. Mothers should consume FA daily and before pregnancy and from both natural and complementary sources in order to optimize the enhancement of maternal and fetal health. Further action should be taken by Saudi health organizations in empowering mothers to take an adequate source of FA at the appropriate time and frequency.

## References

[1] Cieślik E, Cieślik I (2018). Occurrence and significance of folic acid. Pteridines.

[2] Babgi MA, Al-Jifree HM, AlShehri OA, Khan MA, Khogeer AN, Alqurashi MA (2019). Awareness of risk factors and preventive measures for neural tube defects: perception towards pregnancy termination in the Saudi population. J Neonatal Perinatal Med.

[3] Kurdi AM, Majeed-Saidan MA, Al Rakaf MS, AlHashem AM, Botto LD, Baaqeel HS, Ammari AN (2019). Congenital anomalies and associated risk factors in a Saudi population: a cohort study from pregnancy to age 2 years. BMJ Open.

[4] Barchitta M, Maugeri A, Magnano San Lio R, Favara G, La Mastra C, La Rosa MC, Agodi A (2020). Dietary folate intake and folic acid supplements among pregnant women from southern Italy: evidence from the “Mamma & Bambino” cohort. Int J Environ Res Public Health.

[5] Pelletier Pelletier, J. L., Reilly Reilly, R. R., & Bigornia, S. (2017) (2017). Assessment of the intake and knowledge of folate/folic acid among UNH students. https://scholars.unh.edu/honors/376/.

[6] McWalter P, Al Shmassi A, Eldali A (2015). Awareness and use of folic acid in a clinic-based Saudi pregnant population. Saudi J Med Med Sci.

[7] Alblowi SA, Alomayri MH (2018). Assessment of knowledge, awareness and behavior of folic acid use among females during the childbearing period in Tabuk City, 2017. Egypt J Hosp Med.

[8] Al-Holy M, Eideh A, Epuru S (2013). Awareness of folic acid intake among women in the childbearing age in Hail region—Saudi Arabia. 2013.

[9] Ahlam Al-Zahrani, Al-Marwani S (2022). The effectiveness of an educational session about folic acid on pregnant women's knowledge in Yanbu City, Kingdom of Saudi Arabia. AIMS Med Sci.

[10] Hisam A, Rahman MU, Mashhadi SF (2014). Knowledge, attitude and practice regarding folic acid deficiency; a hidden hunger. Pak J Med Sci.

[11] Ahmad B, Anam N, Khalid N, Mohsen R, Zaal L, Jadidy E, Alalati S (2013). Perceptions of women of reproductive age about vitamin and folic acid supplements during pregnancy, Taibah University, Almadinah Almunawwarah, Kingdom of Saudi Arabia. J Taibah Univ Medical Sci.

[12] Al-Hakeem M (2012). Impact of education on knowledge and use of folic acid among Saudi women. Pak J Med Sci.

[13] Yadav V, Dabar D, Prasad P, Patel NS, Agarwal SS (2020). Does India need 'mandatory flour fortification with folic acid' policy to prevent neural tube defects?. J Family Med Prim Care.

[14] Li D, Huang L, Yang W (2019). Knowledge, attitude and practice level of women at the periconceptional period: a cross-sectional study in Shaanxi China. BMC Pregnancy Childbirth.

[15] Omobolanle OA, Olaitan AO, Rukayat OO (2019). Folic acid for the prevention of fetal neural tube defects. Afr J Nurs Midwifery.

[16] Lassi ZS, Salam RA, Haider BA, Bhutta ZA (2013). Folic acid supplementation during pregnancy for maternal health and pregnancy outcomes. Cochrane Database Syst Rev.

[17] Prabahar KK, Alenazi TS (2020). Role of pharmacist's counseling on folate compliance. Saudi Journal for Health Sciences.

[18] Alqahtani A, Alanazi AB, Alduhayan S (2019). Adherence of Saudi women with epilepsy to folic acid intake in pre-conceptional period in Riyadh, Saudi Arabia. Neurology.

[19] Wegner C, Kancherla V, Lux A (2020). Periconceptional folic acid supplement use among women of reproductive age and its determinants in central rural Germany: results from a cross sectional study. Birth Defects Res.

[20] Alfawaz HA, Khan N, AlOteabi N, Hussain SD, Al-Daghri NM (2017). Factors associated with dietary supplement use in Saudi pregnant women. Reprod Health.

[21] Hisam A, Rahman MU, Mashhadi SF (2014). Knowledge, attitude and practice regarding folic acid deficiency; a hidden hunger. Pak J Med Sci.

[22] Alodan AA, Ghoraba MA (2018). Maternal knowledge and use of folic acid among Saudi women attending antenatal care clinic at Security Forces Hospital, Riyadh, Saudi Arabia. IOSR Journal of Nursing and Health Science.

